# Variants of uncertain significance in the era of high-throughput genome sequencing: a lesson from breast and ovary cancers

**DOI:** 10.1186/s13046-020-01554-6

**Published:** 2020-03-04

**Authors:** Giulia Federici, Silvia Soddu

**Affiliations:** Cellular Networks and Molecular Therapeutic Targets Unit, Department of Research and Advanced Technologies, IRCCS-Regina Elena National Cancer Institute, Via Elio Chianesi 53, 00144 Rome, Italy

**Keywords:** Next-generation sequencing, Germline and somatic mutations, Variant classification, Variants of uncertain significance, Functional tests, ATM gene

## Abstract

The promising expectations about personalized medicine have opened the path to routine large-scale sequencing and increased the importance of genetic counseling for hereditary cancers, among which hereditary breast and ovary cancers (HBOC) have a major impact. High-throughput sequencing, or Next-Generation Sequencing (NGS), has improved cancer patient management, ameliorating diagnosis and treatment decisions. In addition to its undeniable clinical utility, NGS is also unveiling a large number of variants that we are still not able to clearly define and classify, the variants of uncertain significance (VUS), which account for about 40% of total variants. At present, VUS use in the clinical context is challenging. Medical reports may omit this kind of data and, even when included, they limit the clinical utility of genetic information. This has prompted the scientific community to seek easily applicable tests to accurately classify VUS and increase the amount of usable information from NGS data. In this review, we will focus on NGS and classification systems for VUS investigation, with particular attention on HBOC-related genes and in vitro functional tests developed for ameliorating and accelerating variant classification in cancer.

## Background

The advent of gene sequencing, by Sanger’s method first [1], then followed by more advanced technologies, has clarified the role played by genetic mutations in inherited disorders, such as rare diseases, and common pathologies, such as multifactorial diseases, leading to their molecular definitions. From the very beginning, this molecular characterization has shown its great potential in the clinic for its diagnostic and, subsequently, therapeutic implications. This potential was enhanced with the advent of high-throughput NGS methodologies [2]. NGS has enormously increased the sensitivity and the efficacy of genetic testing for both Mendelian diseases and multifactorial disorders by enabling the definition of gene causality for rare diseases and the genetic background in neoplastic malignancies [2, 3]. It is now broadly and routinely used into research and clinical practices, to ameliorate both basic knowledge and clinical patient management. Indeed, as a result of NGS and computational progress, we can now benefit from a large amount of information concerning genetic variants present in the general population or in specific pathological subgroups. This is of particular relevance for oncology diseases, for which NGS has identified patient subgroups for prevention strategies and precision treatment, unveiling many different kinds of variants in numerous genes [2].

Genetic testing for cancer risk assessment started in the 1990s, as many clinicians and geneticists began to take into account genetic variants found in cancer patients and family members. Being the most important predisposing genes in female malignancies, BRCA1 and BRCA2 were among the first cancer genes undergoing full sequencing analysis starting from 1996 [4, 5]. Nowadays, high-throughput genetic testing has led to the identification of many other genes related to tumor risk or therapy response. This growing knowledge has been paralleled by constant advances in technology and bioinformatics, allowing even more precise use of gene sequencing. However, as the quantity and complexity of genetic information increase, the number of VUS rises as well, leading to an unavoidable necessity of standardizing their interpretation and classification [6].

The aim of this review is to describe VUS implications in clinical oncology, with a particular attention to HBOC-related genes, and the functional analyses developed to help VUS classification and better exploit NGS information.

### The next-generation sequencing paradox

NGS is a massive parallel sequencing technology that permits simultaneous sequencing of hundreds of DNA/RNA fragments [7]. This technology allows the analysis of single gene- or multi-gene panels enriched for the disease-related genes of interest. Compared to classical standard sequencing methods, NGS simplifies, accelerates, and enlarges the number and variety of sequences analyzed. Indeed, it is possible to sequence different regions (exons and introns), whole exomes (protein-coding regions), or whole genomes, increasing the retrieved information but keeping the costs contained [7]. The choice of the right strategy for genetic testing (e.g., single- or multi-gene panels) and methodology depends on several factors, which include medical purpose (e.g., kind of disorder, etiology, family history), technical considerations and costs [3]. All these issues have been extensively and exhaustively described in many articles that report technical specifications and advances, sequencing purpose, methodological and clinical advantages and relative pitfalls, and we refer to these publications for more detailed information [3, 7–10].

In oncology, for which research, diagnosis, and therapy demands are more heterogeneous than for monogenic diseases, two additional distinctions are taken into consideration: the genetic testing for germline mutations in cancer predisposition genes, in the diagnosis of hereditary cancer forms, and the genetic testing for somatic mutations in “actionable” genes for therapy evaluation, in sporadic tumors.

In addition to the remarkable benefits brought by the advent of NGS in cancer investigation, this technology has created a paradoxical relative shortage of answers in the face of the massive quantity of information generated by high-throughput technologies [11, 12]. The identification of known somatic or germline mutations accelerates diagnosis, development, and use of drugs that specifically target tumor-driving mutations. Indeed, the use of sequencing data ameliorates patient outcomes by improving the identification of targetable mutations and enlarging the number of patients eligible for specific therapies [11]. However, there are no available drugs for many known mutations or newly discovered variants, yet. While NGS is revealing deep tumor molecular characterization, it may not be sufficient to improve patient outcome or treatment, e.g., in the absence of actionable mutations [12]. Furthermore, NGS has accelerated the identification of many diverse diagnostic sub-groups of patients, but studying populations sufficiently large to be statistically significant is still challenging. This makes rare variants difficult to use for clinical purposes, such as the performance of controlled clinical trials [11]. Another possible challenge in NGS use is the time it may take to receive sequencing results, which might be too long to match clinical needs [11]. Although NGS costs have dramatically decreased in absolute term, this technology is not always financially sustainable in all research and clinical environments [12]. Due to the great number of variants obtained, NGS data are complex and uncertainty may arise in physicians interpreting the results [11, 13]. Moreover, in the last decade, the scientific community has faced the definition of an increasing number of variants that cannot be categorized into strict and clear classifications [6]. The ever-growing accumulation of genetic data generates larger and larger percentages of VUS, and this is especially true of oncological diseases, for which large gene-panel sequencing is often required [6, 14]. Indeed, pathogenic variants often represent only a small percentage (< 20%) of all the variants established in high risk cancer genes, such as BRCA1/2 [15]. A summary of benefits and possible pitfalls related to the use of NGS is reported in Table 1.

**Table 1:** The NGS Paradox at a glance: a list of benefits and possible pitfalls

BENEFITS	PITFALLS
✓ High-throughput technology progress	✓ Great challenge for clinical use
✓ Use and development of targeted drugs	✓ New mutations with no drugs
✓ Broad tumor characterization	✓ Uncertainty in patient outcome
✓ Identification of many diagnostic sub-populations	✓ Many small sub-populations with poor statistical power
✓ Simultaneous gene sequencing	✓ Time consuming
✓ Cost decrease	✓ Not always accessible
✓ Growing number of data	✓ Difficult data interpretation and classification
✓ Many newly discovered variants	✓ Increase of VUS

### Guidelines for variant classification

Historically, inherited disorders were diagnosed by direct evidence, such as the phenotype, the segregation of the variant with the disease, or personal and family history. With the advent of gene sequencing and the exponential expansion of variants, the requirement for variant classification became essential [16]. Guidelines to define variant pathogenicity in inherited disorders and, later, for somatic and VUS classification were established. Among the first recommendations, there are those by the American College of Medical Genetics (ACMG), that provide five categories of interpretation based on variant reporting and disease association for Mendelian diseases [17–20]. In addition, they refer to the Association for Molecular Pathology (AMP) workgroup for the interpretation guidelines of somatic variants [16, 21, 22]. Also the International Agency for Research on Cancer (IARC) system subdivides gene variants into 5 classes: classes 1 and 2 include benign and likely benign variants, respectively. Classes 4 and 5 represent pathogenic and likely pathogenic variants, respectively. Class 3 includes VUS (Table 2) [5, 15]. The IARC states that it is essential to discriminate between variants with scarce information (class 3) and variants with strong but not undeniable evidence of disease association (classes 2 and 4) [5, 15]. Class 3 is the most numerous one, comprising about 40% of all variants discovered thus far [23].

**Table 2:** IARC classification system for genetic variants

Class	Description	Probability of being pathogenic (5)	Counseling consequences
1	Benign	< 0.001	Consider as “no mutation detected”
2	Likely benign	0.001–0.049	Consider as “no mutation detected”
3	Uncertain	0.05–0.949	Survey depending on family history
4	Likely pathogenic	0.95–0.99	Ascertained high risk regimen
5	Pathogenic	> 0.99	Ascertained high risk regimen

Concerning models for VUS classification, great efforts have been made by the IARC Unclassified Genetic Variants Working Group in concert with other groups, such as the Breast cancer Information Core (BIC) for BC predisposition genes [24]. VUS classification is largely independent of the occurrence of the variants in the germline or the soma, and irrespective of the disease being inherited or sporadic. *BRCA1* and *BRCA2* VUS classifications have been used as models for other kinds of VUS. In 2004, Goldgar and collaborators brought together different sources of evidence, such as frequency in case versus controls, co-occurrence with a known deleterious mutation, co-segregation with the disease in families, occurrence of disease in relatives, and biochemical evidence, such as residue position, conservation and functional assays [24]. The combination of these data can determine the odds of causality by calculating the posterior probability that each variant is pathogenic [24]. In 2008, the *Human Mutation* journal published a special issue with the title “Assessing mutation pathogenicity in cancer susceptibility genes”. This issue collects many articles curated by the IARC that deeply present and explain all the controversies in and solutions to VUS classification [5, 25–30]. Briefly, the IARC provides standards for the classification of VUS in high-risk cancer susceptibility genes [5, 25]. This system is based on both direct and indirect evidence, as stated in [24] and, in addition to the elements listed above, takes advantage of the likelihood ratio model calculated for *BRCA1/2* in order to use systematically all the available information in a quantitative way [26, 31]. Such information includes tumor pathological characteristics [28], variant functional effects [27], in silico analysis based on sequence-alignment methods, as in missense variant investigation [30].

It is evident that a correct genetic variant classification is essential for managing the genetic information obtained. In some cases, for example, it is mandatory to associate a variant to the correct pathological definition, since it correlates with a specific therapeutic or preventive treatment.

The development of these models and guidelines required the formation of curated databases that integrate as much information as possible. It is in this context that in 2013 the Clinical Genome Resource (ClinGen) project has been launched to create a central resource that defines the clinical validity, the pathogenicity and the clinical usefulness of the genomic information [32]. A clear example of the utility of this resource is represented by the work of Lee and collaborators [33]. Analyzing the HBOC-related genes, the authors defined *BRCA1/2* as the only genes with a “definitive” assertion for predisposition to both BC and OC. Instead, *ATM*, *BARD1*, *CDH1*, *CHEK2* and *PALB2* have a “definitive” association only to BC, while *BRIP1*, *RAD51C* and *RAD51D* only with OC [33]. A key source derived from the ClinGen project is the popular database ClinVar, which archives information on variants with clinical interest [29, 32, 34]. We refer to the next sections for further description.

### Variant clinical use: many sides of the same coin

Two different aspects have to be taken into consideration when dealing with the clinical use of variants and VUS in particular: variant investigation and clinical management.

#### Variant investigation

This first aspect concerns the procedures necessary to obtain the classification data. Many databases help geneticists and clinicians in the interpretation of gene sequencing data, maximizing the information for standard patient care. In 2015, Richards and colleagues highlighted the existence of many databases, recommending a careful use of these tools [18]. The authors distinguished four main types of database: *i)* population databases with data concerning the frequency of variants within healthy and diseased populations, *ii)* disease databases that collect variants, on the basis of known clinical evidence found in patients and derived from bibliographic references or clinical laboratory/industry submissions, *iii)* sources of genome reference sequences, and *iv)* in silico predictive tools that use different algorithms, to determine variant consequence at the nucleotide or amino acid level (e.g., protein sequence modification or splicing sites alterations). These in silico tools are based on sequence alignment and evolutionary conservation, location, and biochemical evaluation of substituted residues. These computational programs may use one or a combination of these criteria and may vary in specificity and sensitivity, attaining 65–80% accuracy when investigating missense variant predictions with known disease implications [18, 35]. A list of the most commonly used databases and tools for germline and somatic variant investigation is shown in Table 3 for disease and population databases and Table 4 for in silico predictive algorithms.

**Table 3:** Examples of disease and population databases

Tools	Website	Description
ClinVar	https://www.ncbi.nlm.nih.gov/clinvar/	Freely accessible, public archive of reports of the relationships among human variations and phenotypes, with supporting evidence.
ClinVar Miner	https://clinvarminer.genetics.utah.edu/	Interface for viewing ClinVar data. Complements the existing ClinVar database by enabling exploration of the data at different levels of granularity and from different perspectives. Statistics for current data and for historical data can be viewed relative to all submissions, submitters, conflicting submissions and genes.
dbSNP	https://www.ncbi.nlm.nih.gov/snp/	Public-domain archive for a broad collection of simple genetic polymorphisms. This collection includes single-base substitutions, small-scale multi-base “indels”, and retroposable element insertion and microsatellite repeat variations.
Leiden Open Variation Database (LOVD)	http://www.lovd.nl/3.0/home	Flexible, freely available tool for gene-centered collection and display of DNA variants.
Cosmic	https://cancer.sanger.ac.uk/cosmic	Source of expert manually curated somatic mutation information relating to human cancers.
cBioPortal	https://www.cbioportal.org/	Open-access, open-source resource for interactive exploration of multidimensional cancer genomics datasets. Stores non-synonymous mutations, DNA copy-number data, mRNA and microRNA expression data, protein-level and phosphoprotein level data, DNA methylation data, and de-identified clinical data.
BRCA Exchange	https://brcaexchange.org/	Open-access web portal resource to display *BRCA1/2* variants drawn from global sources and to enable *BRCA1/2* variants to be expert reviewed, interpreted, classified, and aggregated in an integrated data system. The publicly accessible display of these classifications, with supporting evidence, facilitates accurate understanding of the clinical relevance of any individual *BRCA1/2* variant.
GnomAD	https://gnomad.broadinstitute.org/	Resource with the goal of aggregating and harmonizing both exome and genome sequencing data from a wide variety of large-scale sequencing projects, and making summary data available.
Exome sequencing project	https://evs.gs.washington.edu/EVS/	Database for discovering novel genes and disease mechanisms by pioneering the application of NGS of the protein coding regions of the human genome across diverse, richly-phenotyped populations.
1000Genomes	https://www.internationalgenome.org/	Public catalogue of human variation and genotype data for inferring a large complement of variants, including “indels” and structural variants, in panels of people worldwide for whom only a small subset of SNPs have been analyzed, using partial sequencing techniques such as genotyping arrays.
Human Gene Mutation Database	http://www.hgmd.cf.ac.uk/ac/index.php	Up-to-date and comprehensive reference source to the spectrum of inherited human gene lesions. It includes the first example of all mutations causing or associated with human inherited disease, plus disease-associated/functional polymorphisms reported in the literature.
Human Genome Variation Society	https://www.hgvs.org/	Source for the discovery and characterization of genomic variations including population distribution and phenotypic associations, by promoting collection, documentation and free distribution of genomic variation information and associated clinical variations.

“indel” = insertion and/or deletion. Database and algorithm descriptions were taken from respective websites.

**Table 4:** Examples of in silico algorithms

Tools	Website	Description
PolyPhen-2	http://genetics.bwh.harvard.edu/pph2/	Predicts possible impact of an amino acid substitution on the structure and function of a human protein using straightforward physical and comparative considerations.
Provean	http://provean.jcvi.org/seq_submit.php	Predicts whether an amino acid substitution or “indel” has an impact on the biological function of a protein.
Sift	https://sift.bii.a-star.edu.sg/www/SIFT_seq_submit2.html	Predicts whether an amino acid substitution affects protein function based on sequence homology and the physical properties of amino acids.
Mutation taster	http://www.mutationtaster.org/	Predicts the functional consequences of amino acid substitutions, intronic and synonymous alterations, short “indel” mutations and variant spanning intron-exon borders.
Mutation assessor	http://mutationassessor.org/r3/	Predicts the functional impact of amino-acid substitutions in proteins, such as mutations discovered in cancer or missense polymorphisms. The functional impact is assessed based on evolutionary conservation of the affected amino acid in protein homologs.
FATHMM	http://fathmm.biocompute.org.uk/	High-throughput web-server capable of predicting the functional consequences of both coding variants, i.e., non-synonymous single nucleotide variants (nsSNVs), and non-coding variants through Hidden Markov Models.
Align-GVGD	http://agvgd.hci.utah.edu/agvgd_input.php	Freely available, web-based program that combines the biophysical characteristics of amino acids and protein multiple sequence alignments to predict where missense substitutions in genes of interest fall in a spectrum from enriched deleterious to enriched neutral.
Human splicing finder	http://www.umd.be/HSF3/HSF.shtml	Predicts the effects of mutations on splicing signals and identifies splicing motifs in any human sequence. It contains all available matrices for auxiliary sequence prediction to identify exonic and intronic motifs.

“indel” = insertion and/or deletion. Database and algorithm descriptions were taken from respective websites.

Many issues concerning data storage and interpretation have arisen with the accumulation of large amounts of genetic data. The IARC classification system is routinely used in clinical practice, but constant updating of classified variants is needed. In general, there is a need to simplify and standardize genetic information input/output, definition, and interpretation among different platforms. Indeed, information on specific variants can be found only in one computational platform, while, in other cases, the same variant is reported in different platforms but with different, even divergent class definitions, due to review status, criteria provided, interpretations submitted, and the criteria chosen for sequence/conservation-based analysis [18]. Another great concern is about data handling: genomic data availability would benefit from encouraging data sharing, long-term database update, and increased economic and scientific support [18, 25]. Many websites exist, but only few of them are updated to the latest genetic, population and reference information, some progressively run out of funds, others are not publicly open or user-friendly.

#### Clinical management

The second aspect to consider is the clinical practice: VUS may be omitted from medical reports due to lack of clinical and functional information about them. Only already established pathogenic variants are often reported and considered in standard clinical processes, necessarily leaving outside genetic information of still unknown significance. This leads to the underestimation of patients’ genetic data and, possibly, creates confusion in patients when receiving notice of undefined variants [13, 15, 36, 37]. Furthermore, Capoluongo and colleagues brought to the attention another issue: the different consideration given to somatic and germline mutations and their testing. Indeed, the scientific community still debates about the blood-to-tumor (germline to somatic) or tumor-to-blood (somatic to germline) test benefits and limitations, which depend on the gene and the mutation under investigation, sample DNA enrichment, time consumption, and financial resources [38]. Although somatic tissue mutations are the ultimate drivers of cancer development and the direct markers for targeted therapy, germline sequencing directs early patient management and predicts treatment sensitivity or resistance. This implies clinicians have to choose between germline and somatic testing, depending on the case they are dealing with, evaluating what is not informative or economically affordable.

In the last few years, many authors have reported advantages and disadvantages of using reference clinical databases. For example, the importance of “fixed” and “flexible” systems that, on the one hand, set standard guidelines and on the other adapt over time to new knowledge. It has become evident that the use of integrated databases including both clinical and functional evidence should be preferred. Such databases correlate disease frequency and the molecular consequences of a specific variant on the affected protein product [39]. At present, due to insufficient in vitro test availability, variant classification is influenced more by clinical observations than by the effects of gene alteration on protein function [19, 40]. Still, the use of integrated databases would help the establishment of universal rules for variant classification and terminology.

Given the growing number of *BRCA1* and *BRCA2* VUS, their organized collection appeared immediately mandatory. For this reason, in 2009 the Evidence-based Network for the Interpretation of Germline Mutant Alleles (ENIGMA) international consortium became established (https://enigmaconsortium.org/) [41]. ENIGMA is a wide research-based collaboration with the aim of providing methods to facilitate specifically the classification of the *BRCA1* and *BRCA2* genes and of other BC susceptibility genes. For this purpose, the consortium provides the criteria for assessing variant significance based on multifactorial likelihood models that include population and clinical evaluations and bioinformatic predictions, and promotes data sharing of large-scale projects with variant annotations [26, 42]. Over time, the ENIGMA variant classification data have been collected in the global BRCA Exchange database, together with data from other clinical and population databases (e.g., ClinVar, LOVD, GnomAD), to provide updated and revised reports of variant interpretations [43]. The ENIGMA approach has been exemplary for HBOC-related gene classification for clinical utility. It has been used as a model for the implementation of genetic testing and the development of additional variant investigation and classification strategies in other tumor-related genes [41, 44].

To attain the most accurate variant classification, panels of experts including clinicians, medical geneticists, pathologists, computational and molecular biologists actively encourage the establishment of dedicated medical boards in the clinical practice [11, 12]. This recommendation is improving the collection of clinical information, family history, sequencing data, and functional experimental results, with the goal of providing patients with enhanced counseling. It is also encouraging variant reports, improving pathological classification, and promoting the sharing of the obtained evidence. The optimization of genomic data management will ameliorate and increase reference clinical databases and will help to reclassify orphan variants into specific pathological classes. This is especially important when dealing with VUS of “actionable” genes, for which the combination of direct (co-occurrence, co-segregation, family history) and indirect (in silico/bioinformatic analysis and functional assays) approaches would support their classification and consequently their use in the clinical practice [23, 40].

### VUS: definition and consequences

One of the main databases currently used by the scientific and clinical community worldwide is ClinVar (https://www.ncbi.nlm.nih.gov/clinvar) [34]. It is an international archive of variant-condition interpretations hosted by the NCBI, based on a query-search engine technology and it represents a wide genetic data collection, allowing single variant/gene exploration, interpretation, and sharing [34]. Furthermore, to better understand the impact of genome sequencing on variant definition and achieve a wider perspective, a working group from Utah University developed the ClinVar Miner website (https://clinvarminer.genetics.utah.edu) [45]. It provides a thorough overview of all genetic variants and it is currently updated to December 2019 with about 1000,000 submissions from ClinVar on more than 670,000 variants. ClinVar Miner recapitulates the great diversity of variants in quantity and quality, for more than 30,000 genes, highlighting three important characteristics: *i)* the genes with the highest number of submitted variants include the most relevant tumor risk genes, such as *BRCA1* and *BRCA2*, *APC*, mismatch repair genes, and *ATM* (Table 5), *ii)* many submitted variants have conflicting interpretation and/or redundant significance, and *iii)* a large amount of total variants is represented by VUS (Table 6). In addition, the top 10 genes with the highest number of VUS are enriched in tumor-related genes, whose submitted variants are VUS in up to 40–50% of cases (Table 5) [23, 45]. Concerning HBOC-related genes, up to 20% of *BRCA1/2* variants are classified as VUS [46–48].

**Table 5:** VUS in the top 10 genes with the highest number of submitted variants

Gene	Variants	VUS
*TTN*	12,923	7859
*BRCA2*	10,941	5101
*BRCA1*	7614	2913
*APC*	6082	3114
*NF1*	4602	2046
*TSC2*	4563	1446
*ATM*	4266	2361
*MSH6*	4015	2209
*MSH2*	3296	1641
*FBN1*	3228	1011

Data available on the ClinVar Miner website: https://clinvarminer.genetics.utah.edu/

**Table 6:** Number of submitted variants per significance

Submission significance	Variants	Genes
Uncertain significance	266,759	13,346
Likely benign	203,141	9515
Benign	128,364	14,810
Pathogenic	91,322	9998
Likely pathogenic	41,404	4198
Not provided	17,066	1594
Other	2134	109

Data available on the ClinVar Miner website: https://clinvarminer.genetics.utah.edu/

It is clear that ascertained tumor-related genes are the object of massive interest and sequencing evaluation, and this is why they accrue a larger number of data and, consequently, of undefined variants [46–48].

VUS are difficult to classify for three main reasons: *i)* lack of sufficient population-based statistical evidence, *ii)* scarcity of functional evidence, and *iii)* different evaluations by clinicians and researchers. In the first case, VUS might be not so rare, but found in many different pathological conditions and population subgroups, impeding appropriate statistical evaluations and classifications. The second reason is mainly due to the nature of the variant itself: VUS are mainly missense or synonymous substitutions, substitutions of biochemically similar residues, or in-frame insertions/deletions. They may be found in non-coding regions, at less conserved residues, at splicing boundaries, or in less functionally relevant domains compared to true pathological variants. Thus, the impact of such VUS on the proteins and their functions are more difficult to uncover, compared to nonsense mutations. This explains the scarcity and complexity of in vitro assays [15, 40], but strengthens the need for experimental solutions when dealing with VUS. The third reason is due to different approaches taken by scientists and clinicians. For scientists, VUS, non-affecting variants such as polymorphisms, and novel/uncharacterized variants are noteworthy because they can unveil peculiar genetic and protein alterations involved in biochemical processes, although they are not always informative for clinical purposes. On the contrary, medical genetic counselors mostly consider affecting and pathogenic variants with documented involvement in the disease. Furthermore, different laboratories do not necessarily adopt the same standardized reporting format [49]. These divergent approaches create a gap in knowledge and make VUS difficult to use, overlooking potentially disease-relevant information.

In order to improve VUS (re)classification, it has been suggested to introduce genetic testing on family members, while implementing in vitro test development. In this scenario, functional assays, supported by clinical databases and predictive biochemical algorithms, are the most reliable approaches to reach an accurate VUS classification.

### Functional assays to predict variant consequences and significance: evidence from *BRCA1/2*

Through great efforts, several experimental approaches have been developed in the last few years to determine variant functions in pathological processes. In this context, it is important to choose the appropriate model system to recapitulate the biochemical alterations and their biological consequences [40]. Missense substitutions and VUS are more challenging than truncating mutations since, in the former, the effects on protein structure and function can be less evident. Similarly, experimental assessment of enzymatic catalytic domain activity is easier than the evaluation of, e.g., proteins involved in signaling affected by positive and negative feedbacks [40, 46].

The largest part of functional assays developed thus far concerns the study of *BRCA1* and *BRCA2* [50, 51]. The importance of these experiments has raised with the increase in the number of VUS and the advent of personalized therapies. The assignment of specific treatments depending on the mutational profile of the tumor has great impact on the management and prognosis of patients. Variant experimental analysis has led to in vitro treatments with specific agents that have been progressively introduced in the clinical practice. An example are the poly (ADP-ribose) polymerase (PARP) inhibitors (PARPi), which were demonstrated to be effective in breast and ovarian cancers bearing mutations not only in *BRCA1* and *BRCA2*, but also in genes involved in the DNA damage homologous recombination pathway, such as *PALB2*, *RAD51*, *ATM*, *ATR* and *CHK2* [52, 53]. The observation that also other “BRCAness” genes were involved in PARPi sensitivity opened the possibility of investigating other kinds of malignancies with dysfunction in the DNA damage response, where these genes are high-to-intermediate risk factors [54].

Each of these in vitro assays is different from the others, depending on the experimental model, the type of mutation analyzed, the protein region in which it occurs and the function it alters. They range from homology-directed DNA repair assays [55, 56], to in vitro transactivation of specific domains and measurements of ubiquitin ligase activity [57]. Studies with conditional knock-out cells allow to evaluate the capacities of HBOC-related gene variants to rescue lethality, DNA repair, and resistance to PARPi [48, 53, 58, 59]. Other assays focus on micronucleus formation and centrosome amplification in mutated cell lines [60, 61], on the restoration of resistance to damaging agents by *BRCA2* re-introduction [60], or on increased chromosome breakage after γ-irradiation [62]. Variants in non-coding regions, accounting for about 98% of the genome, are acquiring more importance and are mostly classified as VUS. Many studies measure splicing defects by minigene construction or DNA transcript analysis [63, 64], while others are developing assays to investigate interactions among enhancers, promoters or transcription factors [65].

The advent of new genome editing technologies allowed functional testing with amplified potentiality. The direct manipulation of gene sequences allows the analysis of the biological effects of hundreds of single nucleotide variants (SNVs) in specific protein regions by multiplexed experiments. One example of this is saturation genome editing, which takes advantage of the CRISPR/Cas9 technology [66]. Findlay and collaborators improved this system to build a library of homology-directed repair templates with all the possible SNVs in 13 exons of *BRCA1* for simultaneous analysis. They were able to evaluate 3893 SNVs in their native genomic context and assigned a functional score, predictive of pathogenicity. Of all the submitted variants, 72.5% were deemed functional, and 21.1% non-functional. Missense variants were mainly functional (70.6%), but represented the mutational class with the largest percentage of intermediate classification (8.1%), supporting the evidence that missense mutations are the most difficult to define. All these data were confirmed by the high grade of overlapping between the functional score and the ClinVar classification (i.e., 162 out of 169 pathogenic variants scored as non-functional and 20 out of 22 benign variants scored as functional) [67].

These functional assays still share some pitfalls, such as poor feasibility in daily clinical practice. They are strictly dependent on the experimental model, often represented by commercial cell lines that do not recapitulate patient biological processes, and they are time-consuming. Extensive gene editing requires whole research groups and these assays are not applicable to all the different kinds of variant and to every different gene. Nevertheless, they show concrete efficacy in VUS classification and they have directly addressed the need for experimental approaches to the classification of cancer-related gene variants.

### ATM variants and a new possible functional assay: the p53 mitotic centrosomal localization test

One important gene involved in both rare genetic and neoplastic diseases is the *ATM* gene. The acronym derives from Ataxia-Telangiectasia (A-T), a rare autosomal recessive genetic disease caused by biallelic mutations in the *ATM* gene that determine an early-onset disorder characterized by cerebellar ataxia, immunodeficiency and predisposition to cancer [68].

**Fig. 1:**
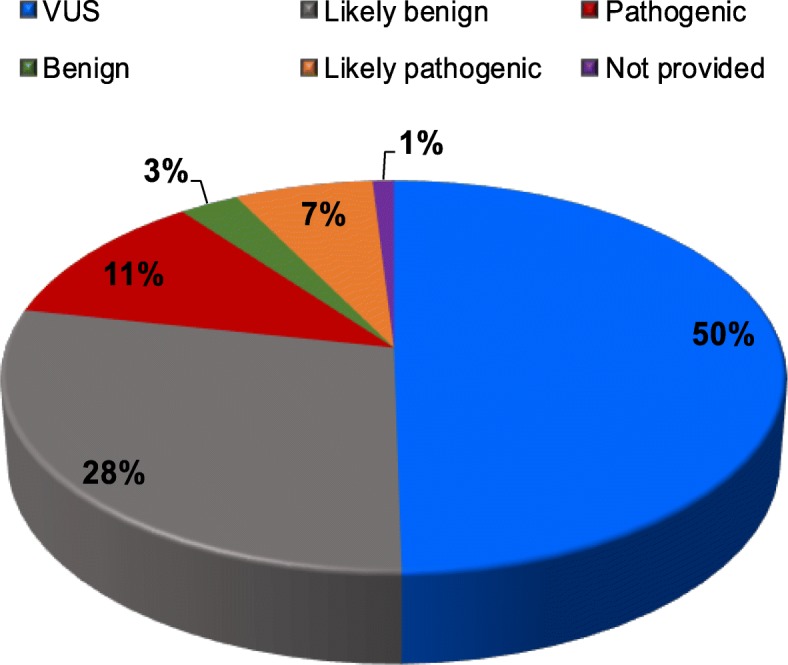
Pie chart representing the percentages of ATM submitted genetic variants subgrouped into clinical classes as reported in ClinVar Miner

The majority of *ATM* mutations causing A-T are nonsense mutations, leading to truncated proteins and severe-to-complete loss of function. *ATM* is a very large gene with many variants, about 40% of which (more than 2000) are represented by VUS (Table 7 and Fig. 1) mostly represented by missense, in-frame, or synonymous mutations.

**Table 7:** Number of ATM submitted genetic variants

Submission significance	Variants
Uncertain significance	2361
Likely benign	1353
Pathogenic	539
Benign	135
Likely pathogenic	312
Not provided	48
Total	4265

Data available on the ClinVar Miner website: https://clinvarminer.genetics.utah.edu/. If a variant has more than one submission, it may be counted in more than one significance column. In this case, the total number of variants will be less than the total of the other cells.

Up to 2% of the whole human population is heterozygous for a pathogenic *ATM* variant and it has been demonstrated that healthy carriers, such as the parents of A-T patients, are more susceptible to tumor formation, with a 5- to 9-fold increased risk of breast cancer in women [69, 70]. Heterozygotes are also sensitive to damage from radio- and chemo-therapy [69–73]. Moreover, it has been demonstrated that rare missense variants of moderate-risk genes, such as *ATM*, confer an increased risk for early-onset breast cancer [74]. Differently from A-T patients, who carry almost exclusively nonsense truncating mutations, cancer patients bear more heterogeneous types of variants. These include out-of-frame mutations with premature stop-codon formation, splicing-affecting variants, synonymous, missense and intronic mutations, with much less evident functional impact, and are enriched in VUS [71, 75]. In the last decade, it has been shown that cell models with *ATM* mutations that cause low or no protein expression are more sensitive to PARPi, supporting clinical implications similar to those of *BRCA1/2* mutant carriers. Such mutations have thus become promising markers for positive PARPi response [76–79]. As a confirmation, a phase II double-blind trial for the treatment of gastric patients with paclitaxel with or without PARPi olaparib showed that ATM^low^ patients receiving the combination of the two drugs had an improved overall survival, compared to the whole population and the group receiving paclitaxel only [80]. Given the role of ATM as an intermediate risk factor for breast, hematological, and pancreatic cancers and as marker of PARPi response, the classification of *ATM* variants is essential for appropriate diagnostic and therapeutic management, and has thus been routinely included in the tumor gene panel for NGS.

To underscore the importance of recognizing clinically-relevant variants, here we highlight how functional analyses may improve *ATM* VUS classification. Our group recently developed a simple functional test based on the ATM-dependent mitotic centrosomal localization of p53 (p53-MCL). This test is able to detect the presence of pathogenic mutant *ATM* in peripheral blood mononuclear cells (PBMCs) [81]. The p53-MCL test takes advantage of the activity of ATM on p53 during mitosis, when ATM phosphorylates p53 at Ser15 and allows its translocation to the centrosomes [82]. This mechanism is important for mitotic surveillance [83] and it has been shown that cells mutated in *ATM*, such as A-T cells, lack this localization [81]. The p53-MCL test is based on an immunofluorescence assay that analyzes the localization of p53 at the centrosomes in PBMCs during mitosis, which is quantitatively impaired in cells carrying pathogenic *ATM* mutations [81]. This was first demonstrated in a large group of A-T patients together with their parents, as obligate healthy carriers of pathogenic mutations, compared with wild-type healthy donors [81]. A preliminary study performed on BC patients carrying germline ATM missense variants or small deletions in both exonic and intronic regions showed that p53-MCL was impaired, highlighting its potentiality for the detection of *ATM* pathogenic mutations also in BC [84]. These data suggest that the p53-MCL test could represent a new functional tool to easily help in the classification of *ATM* VUS in neoplastic diseases.

## Conclusions

In the era of personalized medicine, the use and improvement of NGS has ameliorated the clinical approach and management of patients affected by genetic disorders and neoplastic diseases. The advent of gene sequencing in oncology enhanced the identification of targetable gene mutations, while favoring the administration of existing therapies across different tumor types, depending on their mutational profile. Nevertheless, the discovery of such diverse data in quality and quantity is having enormous implications in the interpretation of genetic variants and their use for therapeutic purposes. The definition of the VUS class of variants prompted clinicians and geneticists to debate how to guarantee maximum standards for patient care. The institution of dedicated tumor medical boards helps to clarify and exploit patient genomic background for diagnostic, prognostic and therapeutic purposes and increases the awareness of the need for integrated systems for appropriate variant classification. The combination of updated disease databases, predictive algorithms and in vitro functional assays is crucial for making VUS eligible for clinical use. For this reason, clinical research is undertaking a great effort to develop in vitro assay to clearly classify VUS.

## Data Availability

All the data reported in this review where downloaded from publicly available databases, as specified along the text.

## Abbreviations

ACMG: American College of Medical Genetics
AMP: Association for Molecular Pathology
A-T: Ataxia-Telangiectasia
BC: Breast Cancer
BIC: Breast cancer Information Core
ENIGMA: Evidence-based Network for the Interpretation of Germline Mutant Alleles
HBOC: Hereditary Breast and Ovary Cancers
IARC: International Agency for Research on Cancer
NGS: Next-Generation Sequencing
OC: Ovary Cancer
p53-MCL: p53 mitotic centrosomal localization
PARP: Poly (ADP-ribose) polymerase
PARPi: Poly (ADP-ribose) polymerase inhibitor
PBMCs: Peripheral blood mononuclear cells
SNVs: Single nucleotide variants
VUS: Variants of Uncertain Significance
